# Transforming University of California, Irvine medical physiology instruction into the pandemic era

**DOI:** 10.1096/fba.2020-00082

**Published:** 2020-12-15

**Authors:** Javier J. Lepe, Arina Alexeeva, Joseph A. Breuer, Milton L. Greenberg

**Affiliations:** ^1^ Department of Neurology University of California Irvine CA USA; ^2^ School of Medicine University of California Irvine CA USA; ^3^ Institute for Clinical and Translational Science University of California Irvine CA USA; ^4^ Department of Physiology and Biophysics University of California Irvine CA USA

**Keywords:** COVID‐19, E‐learning modules, formative assessments, medical physiology, self‐directed learning, undergraduate medical education

## Abstract

At the University of California, Irvine, School of Medicine (UCISOM), the COVID‐19 pandemic is accelerating the transition of face‐to‐face didactic lectures to online platforms. Institutions nationwide have opted to transition their lectures into remote instruction for the upcoming Fall 2020 academic year. UCISOM’s pre‐clerkship Medical Immunology course in the Spring 2020 serves as a template for other medical courses to successfully transform lecture content into virtual presentations. To help facilitate successful large‐scale transition to online courses, UCI developed institutional support and implemented a Division of Teaching Excellence and Innovation (DTEI) Fellowship and iMedEd programs to support medical educators throughout Summer. Previously developed E‐learning modules for renal and acid‐base physiology serve as the foundation for novel pulmonary E‐learning modules at UCISOM. In preparation for the new academic year, in a collaboration between faculty, UCISOM’s top performing second‐year medical students (MS2s) and DTEI fellows worked together during the summer to transition UCISOM’s Medical Physiology and Pathophysiology course online. With over 100 first‐year medical students attending the Medical Physiology course over live synchronous Zoom instruction, formative and summative assessments were incorporated into Canvas modules along with peer‐led review sessions and new E‐learning modules to educate and monitor student progress. The course will maintain existing in‐person active learning activities for students to get hands‐on experience using the latest medical devices while maintaining social distancing. Successful transition to online medical education at UCISOM will depend on increasing use of formative assessments, increased utilization of peer‐led review sessions, and efficient communication to help foster self‐directed learning.

**Key Points**

- The COVID‐19 pandemic is accelerating the transition to online instruction.
- Proper institutional support is essential for medical educators in successful implementation of didactic virtual lectures along with formative assessments to monitor student progress.
- Previously developed E‐learning modules for medical physiology will be expanded in the Fall of 2020.
- Current medical students will expand their role in tutoring first‐year medical students.

## 1 INTRODUCTION

Educating medical students during the COVID‐19 pandemic and maintaining compliance with the ever‐shifting public health guidelines provide unique challenges and opportunities to reform medical education. Improving student learning outcomes involves implementation of innovative remote learning approaches and expanding past successful approaches that focus on virtual instruction. The University of California, Irvine, School of Medicine (UCISOM), Department of Physiology and Biophysics is striving to meet the needs of learners for successful knowledge transfer, while maintaining an interactive environment comparable to that in a face‐to‐face setting.

PHYSIO 543: Medical Physiology and Pathophysiology is a cornerstone course in UCISOM’s first‐year pre‐clerkship curriculum (MS1). In Fall 2020, Medical Physiology will be the largest course taught almost entirely online in UCISOM history. Fortunately, the Medical Physiology course began our transition to online education in 2013 with the implementation of interactive computer‐assisted E‐learning instruction in acid‐base physiology for mobile computer platforms.^1^ Medical students are increasingly using online learning tools including commercially available question banks and teaching videos to supplement in‐person didactic lectures,^2^ and student response to Medical Physiology's E‐Learning approach is largely positive, with the interactive, active learning aspect of the instruction cited as the most important feature.^1^

When compared to traditional in‐person learning, online instruction results in similar outcomes on health professionals’ skills and knowledge.^3^ Remote E‐Learning provides better access to learning resources online, however, the quality of the content and delivery impose significant barriers preventing the effectiveness of E‐Learning instruction.^4^ While COVID‐19 has disrupted traditional graduate medical education (GME) programs,^5^ medical students are enthusiastic for the opportunity to train in these incredible times.^6^ COVID‐19 is also hastening the expansion of the asynchronous learning approaches in undergraduate medical education (UGME).^7^ At UCISOM, the Medical Physiology course is leveraging the lessons learned from pre‐clerkship courses taught in the spring and carefully implementing preparations made in the summer in order to produce an engaging and effective course appropriate for the pandemic era.

## 2 LESSONS LEARNED IN SPRING 2020

PHYSIO 544: Medical Immunology was the first pre‐clerkship course in undergraduate medical education at UCISOM to be taught entirely through remote learning due to the COVID‐19 pandemic, beginning on March 30 and ending May 11, 2020. As such, this course was UCISOM’s and the Department of Physiology and Biophysics’ test lab to determine the best paths to move forward into remote instruction in the pandemic era. Lessons learned from this course are now being analyzed and best practices will be implemented in the upcoming MS1 PHYSIO 543: Medical Physiology and Pathophysiology course scheduled for Fall 2020.

Similar to the other courses in the UCISOM pre‐clerkship curriculum, the Medical Immunology course utilizes diverse teaching modalities, including lecture‐based didactics, small group formative assessments, pre‐exam review sessions, post‐exam review sessions, a self‐directed learning module (SDL), and formal summative assessments. Transition of the course to remote instruction occurred with less than a month's notice and was further complicated by the need to incorporate emerging coronavirus clinical and basic science research into the course, particularly around the immunopathogenesis of COVID‐19.

The course director and lecturing faculty succeeded in the transition to remote instruction. Fortunately, the Department of Physiology and Biophysics have pioneered online instructional modalities, including E‐learning modules and online SDL activities, prior to the COVID‐19 pandemic. In addition, faculty were supported by the UCISOM Division of Educational Technology team to ensure a smooth transition throughout the process. In previous years, SDL activities were entirely online through Canvas, a UCI supported interface learning management system, making this the easiest portion of the course to maintain online. Other sessions, including didactics, formative assessments, pre‐exam, and post‐exam review sessions were held live through the Zoom videoconferencing software. To maintain a glimmer of normalcy, the course sessions were taught synchronously at their previously scheduled times. Zoom links were distributed by email and posted in the Canvas course management software. Lecturing faculty logged into Zoom as a host and presented didactic material via screen share, with students maintaining engagement by logging in at the same time and asking questions by utilizing the chat features for Q&A on Zoom. Small group formative assessments typically involve groups of eight students answering quiz questions related to a previously assigned clinical research article while lecturing faculty migrate through the small groups, clarifying difficult concepts. This year, the formative assessments were maintained and held through Zoom, but the small group format was scrapped. Quiz questions were distributed immediately prior to the scheduled session, and lecturing faculty were available through Zoom to clarify concepts and answer questions prior to quiz submission. Pre‐exam and post‐exam reviews were also held through Zoom, where the lecturing faculty prepared exam‐relevant content and fielded student questions through the Q&A and chat features in Zoom. The course director attended every session, using the Zoom record feature to record and upload the class sessions to Panopto, an online video editing platform, allowing students to review the course material at later dates. Summative assessments occurred at their previously scheduled times and were proctored remotely through the ExamSoft software, relying on the student honor code to fend off potential academic dishonesty. Students were required to sign a statement attesting to knowledge of the honor code and consequences of honor code violation prior to each exam.

The conversion to remote instruction was remarkably successful, both by student evaluations and exam scores. In student evaluations collected following completion of the course, students rated the course at >4.25/5.00 for every evaluation criterium measured, including 4.84/5.00 in the “effectiveness of the course director in running the course,” the highest marks received over the past 5 years. Exam scores had similar distributions compared to the past year, including an average final exam score of 83.0% (compared to 81.2% in 2019), suggesting that the honor code approach to academic honesty was largely successful. Written student feedback discussed how they missed the social interaction with their peers throughout the course, but student narrative feedback was uniformly positive, particularly regarding the incorporation of clinically relevant research related to COVID‐19 pathology and epidemiology. Students acknowledged the efforts involved in adapting to the new conditions of the COVID‐19 pandemic and that the course quality did not suffer despite the transition to remote learning.

## 3 SUMMER 2020 PREPARATIONS

While conversion to online learning in the Spring 2020 semester was largely successful without advanced planning, UCI faculty have used the summer to extensively prepare for teaching in the Fall 2020 semester to accommodate the need for extended online instruction due to the ongoing pandemic. UCISOM guidelines regarding 2020 medical education currently mandate the continuity of remote instruction until January 2021 and may potentially extend guidelines beyond this date. Educational sessions that do not involve a tactile‐learning component, such as lectures and small group discussions, will transition to online, distance‐learning modalities starting in Fall 2020. Activities that involve a tactile‐learning component, including anatomy dissection laboratories and sessions that teach clinical/medical skills, may remain in‐person so long as they adhere to the United States Center for Disease Control and Prevention (CDC) safety guidelines and uphold proper social distancing practices. For such activities, the number of persons per room should be limited to as few persons as practical, ideally to 10 or less persons per learning space. Physical distancing throughout the sessions as well as universal masking or use of face‐shields, as appropriate for the activity, will be enforced. Tactile learning activities will also rely on students and faculty to self‐triage prior each session; individuals with a fever, cough, or any other COVID‐19 symptoms will refrain from attending the activity and suitable learning alternatives will be provided for them.

To comply with newly developed remote learning guidance, UCI has implemented two unprecedented programs to assist the faculty in the transition to remote instruction for fall 2020. First, the main campus Division of Teaching Excellence and Innovation (DTEI) supported and trained graduate students to serve as teaching assistants to assist each basic science course director with the transition to online learning. Second, UCISOM’s Division of Educational Technology implemented the Summer 2020 iMedEd Faculty Development seminar series. Topics included the following: Teaching with Zoom: Overview, Teaching with Zoom: Breakout Rooms, Teaching with Zoom: Other Features, Teaching with Canvas: Discussion Boards, Teaching with Canvas: Group Assignments, Teaching with Podcasts: Panopto, and Inclusive Teaching Considerations. Live faculty development seminars were offered entirely online though Zoom, and these sessions were recorded and provided to all SOM faculty to review at later times.

DTEI, in collaboration with UCI Graduate Division, developed the DTEI UCI Summer Fellowship program to train graduate fellows to assist faculty in developing high quality online courses. Interested UCI graduate students submitted applications detailing their academic field and prior teaching experience to aid in pairing with faculty in similar disciplines. The summer program spanned 10 weeks with an expectation that graduate fellows would dedicate 20 hrs per week for curricular support following an initial 180 hr training workshop. Graduate fellows regularly met with pre‐clerkship course directors throughout the summer to strategize, implement, and test new teaching technologies and resources. In preparations for the Medical Physiology course, MS2 students were included in these meetings to provide valuable input on potential pitfalls and insights into the best paths forward.

Graduate fellows received comprehensive training on several online pedagogical components that could be applied to lectures developed for remote instruction. Lecturers from across the UCI main campus presented training modules over Zoom with a Q&A sessions at the end, familiarizing fellows to Zoom functions. Training topics included pedagogical fundamentals, remote instruction, synchronous and asynchronous remote instruction, and working with faculty. Additionally, training included a review of pedagogical fundamentals such as backward design, student learning outcomes and objectives, active learning techniques, overview of the Family Educational Rights and Privacy Act (FERPA), and most importantly inclusive teaching. All lectures were recorded live and posted online for fellows or instructors to revisit.

The weeklong DTEI fellowship workshop included additional training on third party software to introduce tools to promote interactive E‐learning in a synchronous or asynchronous class setting. Zoom training included establishing administrative controls such as only allowing UCI email accounts to join lecture sessions to avoid Zoom bombing (Zoombies) and generating Zoom polls in advance, saving more time for lectures. Another feature on Zoom that was emphasized was the breakout room option to enhance student interactions by enabling students to work together on class assignments in a remote environment. Graduate fellows were trained on transcription software for conversion of live and pre‐recorded lecture content into text which can be easily integrated into Zoom for students to utilize at their discretion. In addition, fellows were trained on Panopto software and Canvas to familiarize themselves with the functions available for building modules, assignments, quizzes, and integration of pre‐recorded videos.

## 4 PLANS FOR MEDICAL PHYSIOLOGY IN FALL 2020

Medical Physiology is a cornerstone course in the UCISOM pre‐clerkship curriculum and is horizontally integrated with the first‐year histology, anatomy, and clinical foundation courses. In compliance with the guidelines detailed above, the 2020 Medical Physiology course is adjusting our established didactic lectures, active learning sessions, self‐directed learning, peer‐led review sessions, and existing E‐learning content. Lectures will be provided remotely over live‐Zoom during scheduled time slots to provide students with a structured schedule mimicking in‐person curriculum. All Zoom lectures will be recorded as video podcasts and be made available within 24‐hrs for viewing on Canvas. Each lecture will be attended by the course director, in addition to the lecturer, to facilitate student Q&A, and handle any technical problems that may arise during the lecture. Students attending the live sessions will have the opportunity to ask questions via the Zoom‐live chat‐box and those questions will be addressed by either the course director or lecturer throughout the session.

As permitted by current policy, active learning sessions that include hands‐on activity by students can be held in‐person so long as safe‐social distancing practices are maintained in the educational facility. In previous years, medical physiology students participated in an electrocardiogram (ECG) active learning activity, in which they would record their own ECGs using AliveCor KardiaMobile medical devices. As this activity has been demonstrated to be both a valuable and enjoyable learning experience in the past, the course will continue hosting it in the 2020/2021 academic year. The session will be structured as an in‐person, small‐group activity that limits the number of students and faculty per group and maintains social distancing guidelines within each group.

As implemented in previous years in order to satisfy LCME element 6.3 (LCME element 6.3), which states students must participate in some form of self‐directed learning as a means of promoting life‐long learning, students will participate in a “Cardioblog” during the cardiovascular block of medical physiology. Cardioblog utilizes a discussion forum on the Canvas platform that allows students and faculty to both post content and respond to others’ posts. The goal of the Cardioblog is to encourage students to perform a PubMed search on a topic in cardiovascular physiology, read the relevant literature on that topic, and write a short essay on an article of their choosing. Students will then post their essays to the Canvas Cardioblog forum and receive feedback from faculty on their literature searching strategies. Additionally, students will read one of their peers’ posts and respond to it in a manner that provides constructive feedback, a recommended follow‐up reading, or a personal experience that pertains to the original article shared.

The Medical Physiology course, under the initiative of Dr. Kenneth Longmuir, has long been a pioneer in utilizing E‐learning modules to teach pre‐clerkship renal and acid‐base physiology.^1^ For renal physiology, the E‐learning model consists of 11 individual “content modules” of 20–30 slides each. Each content module will present the new subject matter without an audio narration, and includes interactive, active‐learning features such as introductory questions, simple calculations, and pattern recognition. Additional “question and answer modules,” 10–15 questions each, will accompany each of the 11 content modules to provide students with practice questions representative of the degree‐of‐difficulty expected on the renal section exam. The E‐learning authoring software (Trivantis Lectora^®^) offers a variety of question‐and‐answer formats, including true/false, single‐ and multiple‐answer multiple choice, drop‐down list selection, number entry, graphical “hotspot” identification, and drag‐and‐drop graphical manipulation. All question‐and‐answer formats will be utilized and will be important for promoting and maintaining student interest and engagement in the content. Acid‐base physiology will be similarly presented with three content modules and three related question‐and‐answer modules. In addition, three active‐learning case‐study modules will guide students through the acid‐base physiology of asthma, diabetic ketoacidosis, and chloride‐depletion metabolic alkalosis. Each E‐learning module can be completed within an hour's time, and students will have the flexibility to complete the modules at their own pace. To discourage students from waiting to access the E‐learning content immediate prior to the exam, weekly formative assessments (3–4 point quizzes) will be implemented following an in‐person review session that will include in‐class practice problem‐solving exercises (Table 1). Overall, both student exam scores and overall satisfaction with the course material previously administered in this format have been high.^1^

**TABLE 1:** Implementation of renal physiology e‐learning in the medical physiology course.

Week 1	
View E‐learning content modules	View the Q&A modules associated with each subject area
• Body Fluid Compartments	1 hr Zoom review sessions
• Blood Flow and Glomerular	Complete open book quiz. 1 hr time limit on Canvas
• Clearance	
Week 2	
View E‐learning content modules	Zoom review session
• Principles of Transport	Complete open book quiz. 1 hr time limit on Canvas
• Renal Handling of Organics	Same format as week 1
• Renal Handling of Sodium, Chloride, and Water	
• Medullary Osmotic Gradient	
Week 3	
View E‐learning content modules	Zoom review session
• Water Regulation	Complete open book quiz. 1 hr time limit on Canvas
• Salt Regulation	Same format as weeks 1 & 2
• Potassium	
• Hydrogen	
Week 4	
ExamSoft section exam. Closed book. 33 points total.	

The Medical Physiology course will build on the success of the E‐learning approach, and we will transition the respiratory physiology content to a similar E‐learning format for the upcoming Fall 2020 term. Each week, the students will be required to master 3 or 4 subject areas utilizing the content modules and the associated question‐and‐answer modules. Question‐and‐answer modules will be particularly valuable, allowing students to immediately self‐assess, repetitively if needed, their understanding of the content. Toward the end of each week, the instructor will conduct a remote review session that summarizes the content. At the end of each week, students will be asked to complete a 2–3 point open‐book quiz using the Canvas learning management system. We have found that this high‐frequency, low‐stakes testing is, without a doubt, important for ensuring that the students engage in the study of the subject matter on a regular basis, and not procrastinate until shortly before the section exam. Finally, at the end of the renal physiology block, we will administer a comprehensive section exam that certifies to the medical school that the students have successfully mastered the assigned subject matter.

Weekly formative assessments will continue to be administered during renal and acid‐base physiology, and new formative assessments will also be implemented during cardiovascular and endocrine physiology. These new assessments will occur a week before each cumulative block exam (which includes physiology, histology, and anatomy) during both the cardiovascular and endocrinology blocks. The quizzes will be administered remotely utilizing ExamSoft and will be preceded by a faculty‐led review/practice problem session over live‐Zoom. As with the cumulative block exams, UCISOM will rely on the student honor code to uphold academic integrity with remote assessments, and students will be required to sign an attestation prior and during each assessment that lists the academic integrity policy as stated in the UC Irvine School of Medicine Student Handbook. For the final National Board of Medical Examiners (NBME) exam, we will implement the stringent security protocols required by NBME.

The Medical Physiology course has traditionally included in‐person, peer‐led review sessions prior to each organ system‐focused block exam.^8^ The review sessions were held the week before examinations and were led by a team of MS2 students. A few lead tutors would meet with the Physiology course director prior to each review session and discuss high yield concepts for the upcoming exam, after which they would guide the remaining tutors in preparing relevant content review for the MS1 students. Conventionally, the MS1 students would divide into 4–6 groups and rotate between different MS2 tutors, each of which would spend 20 minutes covering a portion of the high yield content. Tutors would begin their session with content presentation, and then, spend the last third of the session going through practice problems and answer MS1 questions about the material. The peer‐review sessions were largely successful, as evidenced by positive student feedback and student preference for peer‐led sessions over faculty led review sessions.

The Medical Physiology course will continue hosting the peer‐led review sessions as described above, however, they will also transition to online platforms. During Summer 2020, two lead tutors were selected by the course director to structure the review sessions, facilitate the transition to remote instruction, and recruit the MS2 content tutors. The lead tutors were provided with a list of the highest scoring MS2 students in the 2019 Medical Physiology course, from which twelve were offered a content tutoring position and two were asked to serve as additional lead tutors. The twelve MS2 content tutors were each assigned two physiology topics: hematologic, neurologic, respiratory, cardiovascular, gastroenterological, endocrine, musculoskeletal, or renal physiology. The four lead tutors, as in previous years, will act as liaisons between the Physiology course director and the twelve content tutors. All communication between the lead tutors, content tutors and course director will occur via Zoom meetings.

To remain adherent to UCISOM 2020 social distancing guidelines, the 2020 MS1 class will be provided with an option for both in‐person and online peer‐led review sessions for each exam review. The online and in‐person review sessions will cover identical material that will be taught by the same set of content tutors. Live‐Zoom will be utilized for the online sessions in order to retain several key advantages of in‐person review: the ability to ask questions and/or clarifications in real‐time, the ability to host discussions between students and tutors, and the ability for students to collaboratively problem solve within breakout rooms. The in‐person sessions will occur at the UCISOM Medical Education Building where students will be divided into rooms with 10 (or less) students with content tutors rotating between rooms. The Medical Education Building has adequate space to host the entire MS1 class in a small group format with maintained social distancing, however, based on previous years’ attendance and the establishment of the online review session we do not anticipate this high of a turnout. At the end of the course, students will be given a second survey where they mark which sessions they attended for each exam (online versus in‐person), explain their reasoning for choosing one modality over the other, and provide feedback about the review session structure. Student responses will be used to gauge the effectiveness of online peer‐led review sessions in comparison to their in‐person counterparts, and thus, improve future transitions to distance learning.

Finally, anticipated structural challenges posed by the pandemic will likely exacerbate underlying educational inequities that the normal UCISOM medical education infrastructure is designed to combat. Students often utilize the institutional resources such as medical libraries, computer access, and high‐speed wireless internet that may not be available at home. These resources will be difficult to obtain while the main campus remains shuttered and the medical campus strictly complies with public health guidelines. Adhering to social distancing guidelines may force students to rely on alternative sources to connect to their online class, which may be limiting. For students to adapt to online instruction, the course director and lecturing faculty will provide constant communication, clarifying expectations and defining course goals. These structural limitations will also make accessibility a critical aspect to ensure all students have access to adequate resources to fulfill course objectives. Using the aforementioned tools and strategies, Medical Physiology at UCISOM is positioned to safely and effectively implement a pandemic‐appropriate curriculum for our future physicians.

## 5 DISCUSSION

There has long been a call for curriculum reform and greater research in UGME,^9^ and COVID‐19 has accelerated the need for teaching innovation and research to transform medical physiology instruction into the pandemic era. A successful medical physiology course will require multiple iterations of planning, curricular implementation, and evaluation of learning outcomes. In Fall 2020, the course will abandon the lecture hall and implement live lecture‐format didactics through Zoom, increased E‐Learning modules,^1^ a single live ECG active learning session, and expanded peer‐led tutorials.^8^ Formative assessments will be utilized throughout the course in the form of quizzes and surveys. Quizzes will be open book, occur more frequently, have more difficult questions, and count for fewer points than previous years. The surveys are intended to give the instructor live student feedback throughout the semester. Other methods of evaluation include continued use of online SDL discussion board assignments that give students the opportunity to engage intellectually to serve as a basis for life‐long learning.

As the new academic year comes to a start, re‐launching Medical Physiology at UCISOM will likely run into potential roadblocks in its infancy. Unforeseen difficulties may reveal themselves despite our extensive preparation. Some of the challenges that may arise include miscommunication between the course director and lecturing faculty, students spending more time than they need to on modules, technical difficulties accessing Zoom or Canvas platforms, and pandemic‐driven exacerbations of underlying educational inequities, including access to stable internet connectivity and adequate study spaces. Although some of these issues can be quickly addressed, it is important for an open line of communication between the course director, faculty, and the students to avoid any delays or confusion on assignments in lectures. There must be a balance between the student's needs and student learning outcomes with specific and well‐defined course objectives mapping to the curricular objectives. With success, first year medical students can have a positive impact in enabling successful remote medical physiology instruction, all the while maintaining the same level of professionalism experienced during in person lectures. During these unprecedented times, UCISOM will strive to uphold the highest of standards of quality medical education with the goal of maximizing student success, and we hope that this manuscript serves as a model for other institutions to follow.
